# Supplementary material for: Discrimination and Characterization of Escherichia coli Originating from Clinical Cases of Femoral Head Necrosis in Broilers by MALDI-TOF Mass Spectrometry Confirms Great Heterogeneity of Isolates
Source: Microorganisms. 2022 Jul 20;10(7):1472. doi: 10.3390/microorganisms10071472 (PMC9323188; doi:10.3390/microorganisms10071472)
Supplement: Supplementary file 1 [file microorganisms-10-01472-s001.zip › microorganisms-1799150-supplementary.pdf]

Table S1: *E. coli* isolates listed according their farm origin (farm no.), their naming (protocol no.), occurrence of femoral head necrosis (yes/no), serotype, PFGE profile, phylogroup. The attribution to the found MALDI-TOF MS clusters is included.

ID	farm no	protocol no.	femoral head necrosis	serotype	PFGE profile	phylogroup	MALDI cluster
**1**	1	16/02297-2 KnoLi 1	no	O2:K1	BR14	B2	3
**2**		16/02297-2 KnoLi 2	no	O2:K1	BR14	B2	3
**3**		16/02297-2 KnoLi 3	no	O2:K1	BR14	B2	3
**4**	2	15/22437-2 KnoRe 1	yes	neg	BR35	A	2
**5**		15/22437-2 KnoRe 2	yes	neg	BR23	D	2
**6**		15/22437-2 KnoRe 3	yes	neg	nt	D	2
**7**	3	15/23061-1 KnoRe 1	yes	neg	BR28	D	2
**8**		15/23061-1 KnoRe 2	yes	neg	BR28	D	2
**9**		15/23061-1 KnoRe 3	yes	neg	BR28	D	2
**10**		16/02376-3 KnoRe 1	yes	O78:K80	BR3	D	4
**11**		16/02376-3 KnoRe 2	yes	O78:K80	BR3	D	4
**12**		16/02376-3 KnoRe 3	yes	O78:K80	BR3	D	4
**13**	4	15/17099-2 KnoRe 1	yes	O2:K1	BR20	A	2
**14**		15/17099-2 KnoRe 2	yes	O2:K1	BR20	A	2
**15**		15/17099-2 KnoRe 3	yes	O2:K1	BR20	A	2
**16**	5	15/23331-2 KnoLi 1	no	neg	BR47	D	2
**17**		15/23331-2 KnoLi 2	no	neg	BR39	D	2
**18**		15/23331-2 KnoLi 3	no	neg	BR47	D	2
**19**		16/00909-1 KnoRe 1	no	O2:K1	BR20	D	4
**20**		16/00909-1 KnoRe 2	no	O2:K1	BR20	D	4
**21**		16/00909-1 KnoRe 3	no	O2:K1	BR20	D	4
**22**		16/00909-1 KnoLi 1	yes	O2:K1	BR20	D	4

**23**		16/00909-1 KnoLi 2	yes	O2:K1	BR20	D	4
**24**		16/00909-1 KnoLi 3	yes	O2:K1	BR20	D	4
**25**	6	15/25176-3 KnoRe 1	no	O2:K1	BR20	D	4
**26**		15/25176-3 KnoRe 2	no	O2:K1	BR20	D	4
**27**		15/25176-3 KnoRe 3	no	O2:K1	BR20	D	4
**28**		16/00911-3 KnoRe 1	no	O2:K1	BR9	B2	4
**29**		16/00911-3 KnoRe 2	no	O2:K1	BR9	B2	4
**30**		16/00911-3 KnoRe 3	no	O2:K1	BR9	B2	4
**31**	7	15/21410-3 KnoLi 1	no	neg	BR10	D	4
**32**		15/21410-3 KnoLi 2	no	neg	BR17	D	2
**33**		15/21410-3 KnoLi 3	no	neg	BR10	D	2
**34**		15/22436-2 KnoRe 1	yes	neg	nt	D	4
**35**		15/22436-2 KnoRe 2	yes	neg	nt	D	4
**36**		15/22436-2 KnoRe 3	yes	neg	nt	D	4
**37**		16/04316-1 KnoRe 1	yes	O78:K80	BR5	A	2
**38**		16/04316-1 KnoRe 2	yes	O78:K80	BR5	A	2
**39**		16/04316-1 KnoRe 3	yes	O78:K80	BR5	A	2
**40**	8	15/20800-1 KnoRe 1	yes	O2:K1	BR20ST1	D	4
**41**		15/20800-1 KnoRe 2	yes	O2:K1	BR20ST1	D	4
**42**		15/20800-1 KnoRe 3	yes	O2:K1	BR20ST1	D	4
**43**		16/00369-2 KnoLi 1	yes	O2:K1	BR20ST1	D	4
**44**		16/00369-2 KnoLi 2	yes	O2:K1	BR20ST1	D	4
**45**		16/00369-2 KnoLi 3	yes	O2:K1	BR20ST1	D	4
**46**		16/02596-3 KnoRe 1	yes	O1:K1	BR26	B1	2
**47**		16/02596-3 KnoRe 2	yes	neg	BR26	B1	2
**48**		16/02596-3 KnoRe 3	yes	neg	BR26	B1	2

**49**		16/02596-3 KnoLi 1	yes	O78:K80	RB38	B1	2
**50**		16/02596-3 KnoLi 2	yes	neg	BR26	B1	2
**51**		16/02596-3 KnoLi 3	yes	O1:K1	BR26	B1	2
**52**		16/03879-3 KnoRe 1	yes	O2:K1	BR14ST1	B2	3
**53**		16/03879-3 KnoRe 2	yes	O2:K1	BR14ST1	B2	3
**54**		16/03879-3 KnoRe 3	yes	O2:K1	BR14ST1	B2	3
**55**		16/03879-3 KnoLi 1	yes	O2:K1	BR14ST1	B2	3
**56**		16/03879-3 KnoLi 2	yes	O2:K1	BR14ST1	B2	3
**57**		16/03879-3 KnoLi 3	yes	neg	BR14ST1	B2	3
**58**	9	15/19461-3 KnoRe 1	yes	O1:K1	BR43	D	2
**59**		15/19461-3 KnoRe 2	yes	O1:K1	BR43	D	2
**60**		15/19461-3 KnoRe 3	yes	O1:K1	BR43	D	2
**61**		15/22114-1 KnoLi 1	yes	O1:K1	BR4	B1	2
**62**		15/22114-1 KnoLi 2	yes	O1:K1	BR4	B1	2
**63**		15/22114-1 KnoLi 3	yes	O1:K1	BR4	B1	2
**64**		15/22748-1 Knoli 1	no	O78:K80	nt	D	2
**65**		15/22748-1 KnoLi 2	no	O78:K80	nt	D	2
**66**		15/22748-1 KnoLi 3	no	O78:K80	nt	D	2
**67**	10	15/23330-2 KnoRe 1	yes	neg	BR7	D	2
**68**		15/23330-2 KnoRe 2	yes	neg	BR7	D	2
**69**		15/23330-2 KnoRe 3	yes	neg	BR7	D	2
**70**		15/24960-2 KnoRe 1	yes	O78:K80	BR32ST1	D	4
**71**		15/24960-2 KnoRe 2	yes	O78:K80	BR32ST1	D	4
**72**		15/24960-2 KnoRe 3	yes	O78:K80	BR32ST1	D	4
**73**		16/03318-3 KnoRe 1	yes	O1:K1	BR19	B2	4
**74**		16/03318-3 KnoRe 2	yes	O1:K1	BR19	B2	4

**75**		16/03318-3 KnoRe 3	yes	O1:K1	BR19	B2	4
**76**		16/03318-3 KnoLi 1	yes	O1:K1	BR19	B2	4
**77**		16/03318-3 KnoLi 2	yes	O1:K1	BR19	B2	4
**78**		16/03318-3 KnoLi 3	yes	neg	BR19	B2	2
**79**	11	15/19578-10 KnoLi 1	no	neg	BR22	A	2
**80**		15/19578-10 KnoLi 2	no	neg	nt	A	2
**81**		15/19578-10 KnoLi 3	no	neg	nt	D	4
**82**		15/24052-3 KnoRe 1	yes	neg	BR8ST1	B2	4
**83**		15/24052-3 KnoRe 2	yes	neg	BR8ST1	B2	4
**84**		15/24052-3 KnoRe 3	yes	neg	BR8ST1	B2	4
**85**		15/24865-1 KnoRe 1	yes	neg	BR1	D	4
**86**		15/24865-1 KnoRe 2	yes	neg	BR1	D	4
**87**		15/24865-1 KnoRe 3	yes	O1:K1	BR1	D	4
**88**		16/02899-1 KnoRe 1	yes	O1:K1	BR2	D	4
**89**		16/02899-1 KnoRe 2	yes	O1:K1	BR2	D	4
**90**		16/02899-1 KnoRe 3	yes	neg	BR2	D	4
**91**	12	15/23060-1 KnoRe 1	yes	neg	BR26ST1	B1	2
**92**		15/23060-1 KnoRe 2	yes	neg	BR26ST1	B1	2
**93**		15/23060-1 KnoRe 3	yes	neg	BR26ST1	B1	2
**94**		15/24413-2 KnoRe 1	yes	neg	BR31	A	2
**95**		15/24413-2 KnoRe 2	yes	neg	BR41	B1	2
**96**		15/24413-2 KnoRe 3	yes	neg	BR31	A	2
**97**		16/02752-3 KnoLi 1	yes	neg	BR20ST2	D	4
**98**		16/02752-3 KnoLi 2	yes	neg	BR20ST2	D	4

**99**		16/02752-3 KnoLi 3	yes	neg	BR20ST2	D	4
**100**	13	15/23332-2 KnoRe 1	yes	neg	BR12	D	2
**101**		15/23332-2 KnoRe 2	yes	neg	BR12	D	2
**102**		15/23332-2 KnoRe 3	yes	neg	BR12	D	2
**103**		16/003317-1 KnoRe 1	no	O78:K80	BR32	D	4
**104**		16/003317-1 KnoRe 2	no	O78:K80	BR32	D	4
**105**		16/003317-1 KnoRe 3	no	O78:K80	BR32	D	4
**106**		16/003317-1 KnoLi 1	yes	O78:K80	BR32	D	4
**107**		16/003317-1 KnoLi 2	yes	O78:K80	BR32	D	4
**108**		16/003317-3 KnoLi 1	yes	O78:K80	BR32	D	4
**109**		16/003317-3 KnoLi 2	yes	O78:K80	BR32	D	4
**110**		16/003317-3 KnoLi 3	yes	O78:K80	BR32	D	4
**111**	14	15/20278-3 KnoRe 1	yes	neg	nt	D	4
**112**		15/20278-3 KnoRe 2	yes	neg	nt	D	4
**113**		15/20278-3 KnoRe 3	yes	neg	nt	D	4
**114**		15/24415-1 KnoRe 1	yes	neg	nt	D	4
**115**		15/24415-1 KnoRe 2	yes	neg	nt	D	4
**116**		15/24415-1 KnoRe 3	yes	neg	nt	D	3
**117**		16/00910-3 KnoRe 1	yes	neg	BR45	B2	2
**118**		16/00910-3 KnoRe 2	yes	neg	nt	A	2
**119**		16/00910-3 KnoRe 3	yes	O78:K80	BR45	B2	2
**120**		16/02374-1 KnoRe 1	yes	O78:K80	BR32ST1	D	4
**121**		16/02374-1 KnoRe 2	yes	O78:K80	BR32ST1	D	4
**122**		16/02374-1 KnoRe 3	yes	O78:K80	BR32ST1	D	4

**123**		16/02374-1 KnoLi 1	yes	O78:K80	BR32ST1	D	4
**124**		16/02374-1 KnoLi 2	yes	O78:K80	BR32ST1	D	4
**125**		16/02374-1 KnoLi 3	yes	O78:K80	BR32ST1	D	4
**126**	15	15/20279-2 KnoRe 1	no	neg	BR34	D	2
**127**		15/20279-2 KnoRe 2	no	neg	BR34	D	2
**128**		15/20279-2 KnoRe 3	no	neg	BR34	D	2
**129**		15/24414-5 KnoRe 1	yes	neg	BR6	D	4
**130**		15/24414-5 KnoRe 2	yes	neg	BR6	D	4
**131**		15/24414-5 KnoRe 3	yes	neg	BR6	D	4
**132**		16/01523-1 KnoLi 1	yes	O2:K1	BR9	B2	4
**133**		16/01523-1 KnoLi 2	yes	O2:K1	BR9	B2	4
**134**		16/01523-1 KnoLi 3	yes	O2:K1	BR9	B2	4
**135**		16/01523-3 KnoRe 1	yes	neg	BR8	D	2
**136**		16/01523-3 KnoRe 2	yes	neg	BR8	D	2
**137**		16/01523-3 KnoRe 3	yes	neg	BR1ST1	D	1
**138**		16/02751-3 KnoRe 1	no	neg	BR1ST1	D	1
**139**		16/02751-3 KnoRe 2	no	neg	BR1ST1	D	1
**140**	15	16/02751-3 KnoRe 3	no	neg	BR1ST1	D	1
**141**		15/24412-3 KnoRe 1	yes	neg	BR29	B2	4
**142**		15/24412-3 KnoRe 2	yes	neg	BR29	B2	3
**143**		15/24412-3 KnoRe 3	yes	neg	BR29	B2	3
**144**	17	15/19580-2 KnoRe 1	yes	O78:K80	BR32ST4	D	4
**145**		15/19580-2 KnoRe 2	yes	O78:K80	BR32ST4	D	4
**146**		15/19580-2 KnoRe 3	yes	O78:K80	BR32ST4	D	4
**147**		15/20949-1 KnoLi 1	yes	neg	BR32ST4	D	4
**148**		15/20949-1 KnoLi 2	yes	neg	BR32ST4	D	4

**149**		15/20949-1 KnoLi 3	yes	neg	BR32ST4	D	4
**150**		15/23207-1 KnoRe 1	no	neg	BR11	A	2
**151**		15/23207-1 KnoRe 2	no	neg	BR40	B1	2
**152**		15/23207-1 KnoRe 3	no	neg	BR27	B1	2
**153**		15/24161-3 KnoLi 1	yes	neg	BR37	A	2
**154**		16/02298-3 KnoRe 1	yes	O2:K1	BR14ST1	B2	3
**155**		16/02298-3 KnoLi 1	no	O2:K1	BR14ST1	B2	3
**156**		16/02298-3 KnoLi 2	no	O2:K1	BR14ST1	B2	3
**157**		16/02298-3 KnoLi 3	no	O2:K1	BR14ST1	B2	3
**158**	18	15/19460-1 KnoRe 1	yes	neg	nt	D	4
**159**		15/19460-1 KnoRe 2	yes	neg	nt	D	4
**160**		15/19460-1 KnoRe 3	yes	neg	nt	D	4
**161**		15/21788-2 KnoLi 1	yes	neg	BR8	B2	4
**162**		15/21788-2 Knoli 2	yes	neg	BR8	B2	4
**163**		15/21788-2 KnoLi 3	yes	neg	BR8	B2	4
**164**	19	15/23732-2 KnoLi 1	yes	neg	BR18	D	4
**165**		15/23732-2 KnoLi 2	yes	neg	BR18	D	4
**166**		15/23732-2 KnoLi 3	yes	neg	BR18	D	4
**167**		16/01362-3 KnoLi 1	no	neg	BR15	B2	3
**168**		16/01362-3 KnoLi 2	no	neg	BR15	B2	3
**169**		16/01362-3 KnoLi 3	no	neg	BR25	B2	2
**170**		16/01362-2 KnoRe 1	no	neg	BR16	B2	3
**171**		16/01362-2 KnoRe 2	no	neg	BR16	B2	3
**172**		16/01362-2 KnoRe 3	no	neg	BR16	B2	3
**173**		16/02598-1 KnoRe 1	yes	neg	BR16	B2	3
**174**		16/02598-1 KnoRe 2	yes	neg	BR36	A	2

**175**		16/02598-1 KnoRe 3	yes	neg	BR16	B2	3
**176**	20	15/20264-3 KnoLi 1	no	neg	BR24	D	4
**177**		15/20264-3 KnoLi 2	no	neg	BR24	D	4
**178**		15/20264-3 KnoLi 3	no	neg	BR24	D	4
**179**		15/23731-2 KnoRe 1	yes	neg	BR18	D	4
**180**		15/23731-2 KnoRe 2	yes	neg	BR18	D	4
**181**		15/23731-2 KnoRe 3	yes	neg	BR18	D	4
**182**		15/24861-3 KnoRe 1	yes	O2:K1	BR13	B2	4
**183**		15/24861-3 KnoRe 2	yes	O2:K1	BR13	B2	4
**184**		15/24861-3 KnoRe 3	yes	neg	BR13	B2	4
**185**		16/01363-1 KnoLi 1	no	neg	BR15	B2	3
**186**		16/01363-1 KnoLi 2	no	neg	BR15	B2	3
**187**		16/01363-1 KnoLi 3	no	neg	BR15	B2	3
**188**	21	15/17098-2 KnoRe 1	yes	neg	BR21	A	2
**189**		15/17098-2 KnoRe 2	yes	neg	BR21	A	2
**190**		15/17098-2 KnoRe 3	yes	neg	BR21	A	2
**191**		15/19104-2 KnoRe 1	no	O2:K1	BR33	B2	4
**192**		15/19104-2 KnoRe 2	no	O2:K1	BR33	B2	4
**193**		15/19104-2 KnoRe 3	no	O2:K1	BR33	B2	4
**194**		15/19103-3 KnoLi 1	yes	O78:K80	BR3	A	2
**195**		15/19103-3 KnoLi 2	yes	O78:K80	BR3	A	2
**196**		15/19103-3 KnoLi 3	yes	O78:K80	BR3	A	2
**197**		15/20799-2 KnoRe 1	yes	neg	BR4	B1	2
**198**		15/20799-2 KnoRe 2	yes	neg	BR4	B1	2
**199**		15/20799-2 KnoRe 3	yes	neg	BR4	B1	2
**200**		15/24599-3 KnoRe 1	no	O78:K80	BR32ST2	D	4

**201**	15/24599-3 KnoRe 2	no	O78:K80	BR32ST3	D	4
**202**	15/24599-3 KnoRe 3	no	O78:K80	BR32ST2	D	4
**203**	15/25396-3 KnoRe 1	no	O78:K80	BR32	D	1
**204**	15/25396-3 KnoRe 2	no	O78:K80	BR32	D	1
**205**	15/25396-3 KnoRe 3	no	O78:K80	BR32	D	1
**206**	15/25396-3 KnoLi 1	yes	O78:K80	BR32	D	4
**207**	15/25396-3 KnoLi 2	yes	O78:K80	BR32	D	4
**208**	15/25396-3 KnoLi 3	yes	O78:K80	BR32	D	4
